# Impact of insecticide-treated bed nets on malaria transmission indices on the south coast of Kenya

**DOI:** 10.1186/1475-2875-10-356

**Published:** 2011-12-13

**Authors:** Francis M Mutuku, Charles H King, Peter Mungai, Charles Mbogo, Joseph Mwangangi, Eric M Muchiri, Edward D Walker, Uriel Kitron

**Affiliations:** 1 Department of Environmental Studies, Emory University, Atlanta, Georgia, USA; 2 Center for Global Health and Diseases, Case Western Reserve University, Cleveland, OH, USA; 3 Centre for Geographic Medicine Research-Coast, Kenya Medical Research Institute, Kilifi, Kenya; 4 Division of Vector Borne and Neglected Tropical Diseases (DVBNTD), Ministry of Public Health and Sanitation, Nairobi, Kenya; 5 Department of Microbiology and Molecular Genetics, Michigan State University, East Lansing, MI, USA; 6 Fogarty International Center, National Institutes of Health, Bethesda MD, USA

## Abstract

**Background:**

Besides significantly reducing malaria vector densities, prolonged usage of bed nets has been linked to decline of *Anopheles gambiae *s.s. relative to *Anopheles arabiensis*, changes in host feeding preference of malaria vectors, and behavioural shifts to exophagy (outdoor biting) for the two important malaria vectors in Africa, *An. gambiae *s.l. and *Anopheles funestus*. In southern coastal Kenya, bed net use was negligible in 1997-1998 when *Anopheles funestus *and *An. gambiae *s.s. were the primary malaria vectors, with *An. arabiensis *and *Anopheles merus *playing a secondary role. Since 2001, bed net use has increased progressively and reached high levels by 2009-2010 with corresponding decline in malaria transmission.

**Methods:**

To evaluate the impact of the substantial increase in household bed net use within this area on vector density, vector composition, and human-vector contact, indoor and outdoor resting mosquitoes were collected in the same region during 2009-2010 using pyrethrum spray catches and clay pots for indoor and outdoor collections respectively. Information on bed net use per sleeping spaces and factors influencing mosquito density were determined in the same houses using Poisson regression analysis. Species distribution was determined, and number of mosquitoes per house, human-biting rates (HBR), and entomological inoculation rate (EIR) were compared to those reported for the same area during 1997-1998, when bed net coverage had been minimal.

**Results:**

Compared to 1997-1998, a significant decline in the relative proportion of *An. gambiae *s.s. among collected mosquitoes was noted, coupled with a proportionate increase of *An. arabiensis*. Following > 5 years of 60-86% coverage with bed nets, the density, human biting rate and EIR of indoor resting mosquitoes were reduced by more than 92% for *An. funestus *and by 75% for *An. gambiae *s.l. In addition, the host feeding choice of both vectors shifted more toward non-human vertebrates. Besides bed net use, malaria vector abundance was also influenced by type of house construction and according to whether one sleeps on a bed or a mat (both of these are associated with household wealth). Mosquito density was positively associated with presence of domestic animals.

**Conclusions:**

These entomological indices indicate a much reduced human biting rate and a diminishing role of *An. gambiae *s.s. in malaria transmission following high bed net coverage. While increasing bed net coverage beyond the current levels may not significantly reduce the transmission potential of *An. arabiensis*, it is anticipated that increasing or at least sustaining high bed net coverage will result in a diminished role for *An. funestus *in malaria transmission.

## Background

Many studies have reported the key role played by insecticide-treated nets (ITNs) in malaria control, especially in sub-Saharan Africa [1]. Their role in reducing human-vector contact and lowering malaria morbidity and mortality is well documented in areas of both high and low endemicity [2-4]. Evaluation of malaria transmission indicators has demonstrated the effectiveness of ITNs in both small- and large-scale programme settings [2,3,5-8]. Whereas untreated bed nets reduce human-vector contact by providing a physical barrier only, treated nets offer both physical and chemical barriers to mosquito-human contact. The chemical barrier operates in three ways: deterrence, excito-repellence and killing [9,10]. Through these effects, bed nets reduce density, feeding frequency, feeding success, and survival of anopheline mosquito vectors [11-13]. By reducing vector densities and survival, ITNs not only decrease malaria exposure for the protected individuals, but also provide protection to the rest of the local human community [14-17] when a particular threshold of bed net coverage is reached [18].

Continuous monitoring of the impact of ITN use on malaria vectors is necessary for sustaining these gains [19]. Prolonged usage of ITNs has been linked to behavioural shifts to exophagy (outdoor biting) for the two important malaria vectors in Africa, *Anopheles gambiae s.l*. and *Anopheles funestus *[20-22]. Other reports indicate that selective pressure due to sustained long term use of ITNs at high coverage resulted in a drastic decline of *An. gambiae s.s*. relative to *Anopheles arabiensis *in western Kenya and in south-eastern Tanzania [13,22]. *Anopheles gambiae s.l*. is a complex of at least seven morphologically indistinguishable species of mosquitoes in the genus *Anopheles*. The complex includes two of the most important and efficient malaria vectors in sub-Saharan Africa; *An. gambiae *s.s. and *An. arabiensis*. With regard to whether sustained ITN use alters host feeding preference of malaria vectors, some studies indicate no change in host selection [23] while others show a slight shift to non-human hosts [24-26].

Bed nets are the most widely used malaria control method in the southern coast districts of Msambweni, Kwale and Kinango (formerly Kwale district), as is the case elsewhere in Kenya. Historically, malaria has been holoendemic along coastal Kenya, with *An. gambiae *s.l. and *An. funestus *serving as the predominant vectors [27]. Net usage on the coast and throughout Kenya was negligible before 2001. Through an assortment of net distribution and delivery channels, net coverage and use has been boosted from 34.2% and 22.4%, respectively, in 2003 [28] to 70.9% and 60.8% in 2008-2009 [29]. The coverage reached > 60% by the end of 2006 following a campaign for mass distribution of free nets in 2006 [30]. A coincidental decline in paediatric hospital admissions for malaria has been reported in all major hospitals along the coast since 2003 [6,31,32]. Most areas in coastal Kenya are now considered to be under low to moderate transmission of 1% - < 20% signaling a possible epidemiological transition from the previously high levels [33].

In anti-malaria programme implementation areas, where every effort has been made to scale up interventions, rigorous vector surveillance remains essential to evaluate the success or failure of such intervention. In the last decade, there have been few entomological studies of vector bionomics in most malaria control areas and none in the three districts studied in this paper. In the study area, the relative contribution to malaria transmission attributable to *An. funestus*, *An. gambiae *s.s., *An. arabiensis *and *Anopheles merus *was 51.0%, 44.7%, 3.8% and 0.5%, respectively, in 1997/1998 [27], when bed net ownership and use was minimal. During the 2009-2010 study period, resting malaria vectors were collected monthly, in eight villages for 21 months by PSC indoors and for 17 months by clay pots outdoors. Using the 1997-1998 data as a baseline, the impact of the introduction of insecticide-treated bed nets on local malaria vectors following several years of high coverage and bed net use, was examined.

## Methods

### Study site and population

**Figure 1:**
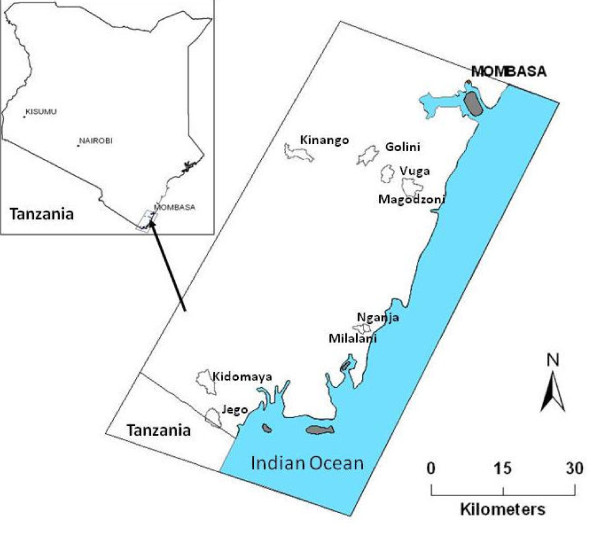
**Map of the study area, Inset: map of Kenya showing the location of study area**.

The 1997-1998 study was conducted in 30 villages; 20 in north coastal Kenya (then Kilifi and Malindi districts and now Kilifi county) and 10 in south coastal Kenya (then Kwale district and now Kwale County which includes Msambweni, Kwale and Kinango districts). The current survey (2009-2010) was conducted in the south coast of Kenya in Msambweni (Msambweni and Vanga divisions), Kwale (Matuga division) and Kinango (Kinango division) districts. Kwale County borders Tanzania to the south-west and the Indian Ocean to the east (Figure 1). The area is hot and humid year round with a range of annual mean temperatures of 23°C-34°C and average relative humidity range of 60%-80%. Altitude ranges from 0 to 464 m above sea level. There are two rainy seasons, April to June and October to November, but some rain falls in nearly every month, especially nearer to the coastline. The total precipitation varies from 900 to 1500 mm per annum along the coastal belt to 500-600 mm in the back country. Malaria is endemic in the study area and the predominant malaria vectors were known to include *An. funestus*, *An. gambiae *s.s. and *An. arabiensis*. More details on the study area are provided in previous publications [27,34].

### Study villages

The 2009-2010 entomological and household survey is part of a large study of the eco-epidemiology of schistosomiasis, malaria and polyparasitism in coastal Kenya (in short the Polyparasitism project). The project covers eight villages grouped by four ecological settings defined by elevation, temperature, rainfall, relief, distance to the Indian Ocean and land cover type (Figure 1): 1) Jego and Kidomaya villages located at the southern tip of Kenya, representing the coastal estuarine environmental setting; 2) Nganja and Milalani villages located near Msambweni district hospital, representing the coastal plain setting; 3) Magodzoni village located at the bottom of the slope, Vuga village located at mid-slope and Golini village located at the top of the slope, representing the coastal slope setting); 4) Kinango village located more inland, representing the inland semi-arid environmental setting. The 1997-1998 survey collected mosquitoes from villages within 2-km radius from 10 selected primary schools in Kwale County. Most of the villages studied in 1997-1998 are very close to those studied 2009-2010 with an overlap in Vuga village [27].

### Mapping of study villages

Specific houses where mosquitoes were collected were not mapped in the 1997-1998 study. However, for each village, the latitude and longitude of the nearby primary school was recorded using a hand-held GPS (Garman International, Inc, Olathe, KS). In the 2009-2010 study, prior to mosquito collection (July to October 2008), the boundary and extent of each village was mapped using a global positioning system (GPS) device (GeoExplorer 2005 series GeoXM; Trimble, Sunnyvale, CA). High resolution Ikonos and quickbird satellite images acquired between 2001 and 2008 were retrieved from GeoEye archives (GeoEye, Dulles, Virginia) for precise mapping of households within the extent of each village as described by Clennon et al., [35]. A GPS (GeoExplorer 2005 series GeoXM; Trimble, Sunnyvale, CA) was used to map newly constructed houses especially in villages where the satellite images archive were more than 4 years old.

### Mosquito collections

In the 1997-1998 study, mosquitoes were collected inside 10 houses within a 2-km radius of each school bi-monthly by PSC from June 1997 to May 1998. The same houses were sampled in each village throughout the study period, although on some occasions, circumstances required substitution by a nearby house. To detect changes in indoor resting between the two study periods, PSC was used to collect mosquitoes in the 2009-2010 study period. Additionally, clay pots which recently were shown to be very efficient in collecting mosquitoes outdoors [36] were used in this study. In the 2009-2010 study, mosquitoes were collected indoors in the eight villages once every 4 weeks by PSC from April 2009 through December 2010, and outdoors using clay pots through August 2010 (discontinued due to poor catch) in 10 randomly selected houses. During the 21-month period, efforts were made to collect mosquitoes in every house in each village, and only rarely were mosquitoes collected from the same house more than once. Mosquito collection by both methods was performed in 10 houses in each of two villages weekly over a period of 71 weeks for clay pots and of 89 weeks for PSC. Mosquito collections were omitted for a week in December 2009 and for 2 weeks in December 2010 during holidays. The collection design was such that mosquito collection was performed in all eight villages within a four-week period. Thus, by the time the use of clay pots were terminated in August 2010, mosquito collection had been performed in 1,420 houses (20 houses in a week for 71 weeks) for the pots and 1,777 houses by PSC (20 houses per week for 89 weeks minus three houses whose owners were absent). Mosquito collection by both methods always started at 06:00 h and ended no later than 10:00 h. For PSC catches of indoor resting mosquitoes, houses were sprayed with 10% pyrethrins dissolved in kerosene using the method described by Gimnig and others [11].

Briefly, white sheets were systematically laid on the entire floor and over the furniture within all the rooms of each house. Then all windows and doors were shut and a mixture of 10% pyrethrins dissolved in kerosene was sprayed on the eaves by a mosquito collector from the outside to prevent the mosquitoes inside the houses from escaping. Inside the house, the walls and roofs were sprayed by another mosquito collector. The houses were then closed for 10-15 minutes in order to knock down the endophilic mosquitoes, after which the knocked down mosquitoes were collected from the white sheets using forceps for each room and placed on moist filter paper inside labeled petri dishes. Outdoor resting mosquitoes were collected during the same morning and in the same 10 houses using clay pots. The clay pots used here were similar to (and procured from the same source as) those used by Odiere et al. [36]). One convectional clay pot with a volume of 20-25 L, an opening of 16-20 cm diameter and a 2-cm-diameter hole placed in the center of the base, was used per house. The night prior to the mosquito collection the pot was placed ≤ 5 metres from each of the 10 houses, preferably at the back of the house close to the bedroom and away from areas with a lot of human activities to avoid disturbing resting mosquitoes. Resting mosquitoes in the pot were recovered using a caged net placed over the pot opening. The pot was then lifted to expose the opening to the light and agitate the resting mosquitoes inside; air is then blown at the bottom into the small hole causing the mosquitoes to take to fly into the caged nets [36]. The captured mosquitoes were aspirated from the caged net into labeled paper cups. Mosquitoes were identified in the field as anophelines or culicines. Mosquito collected by PSC were transported to the laboratory on moist filter paper inside petri dishes and those from clay pots were transferred to the laboratory in the collection cups. Physical presence of a hanging bed net was verified and the total number people who slept in the house the previous night was recorded.

### Larval sampling

In the 2009-2010 study, larval sampling was synchronized with adult mosquito collections such that larval sampling was always done in the same two villages the same week as the adult collections. Larval sampling was started in May 2009. The number of sentinel larval habitats for *Anopheles *mosquitoes varied by village and ranged from three in Nganja village to 19 Jego village. The number of larval habitats sampled during each visit was highly influenced by seasonal rainfall patterns. Sampling was performed using the standard dipper (350 ml dipper) once every 4 weeks in the eight villages. The number of all instars and pupae of anopheline mosquitoes per dipper for a total of ten dips per potential larval habitat was recorded. All third and fourth instar *Anopheles *larvae and pupae were pipetted into polythene bags and transported to the laboratory for morphological identification. The pupae were allowed to emerge and adults were identified morphologically. No larval sampling was performed during the adult mosquito collection in the 1997-1998 study. However, larval sampling was done in 1999 in three out the 10 villages studied in 1997-1998 [34].

### Mosquito processing

Mosquitoes from all catches were sorted and counted in the laboratory at Msambweni District hospital. *An. gambiae *s.l. and *An. funestus *were identified morphologically [37] and their feeding level was classified by abdominal condition. Four categories were represented among female specimens: unfed, fed, semigravid and fully gravid. The 3rd and 4th anopheline larvae were also identified morphologically [37]. All mosquitoes were dried over silica gel and stored in -20°C freezers when completely desiccated. The head and thorax of a portion of the fed and semigravid malaria vectors caught were tested for *Plasmodium falciparum *circumsporozoite protein by enzyme-linked immunosorbent assay (ELISA) [38]. Legs or wings or whole body (depending on availability) of more than half of *An. gambiae s.l*. collected (adults and larvae) were identified to species by PCR as *An. gambiae s.s*. or *An. arabiensis *[39]. To determine host blood meals, abdomens of blood-fed and half-fed mosquitoes were separated from the thorax and head, and nucleic acids extracted from them (DNeasy Tissue Kits, Qiagen). PCR was conducted on these DNA templates in order to amplify a segment of the vertebrate, mitochondrial cytochrome B gene using the "BM" primer pair (5'--CCC CTC AGA ATG ATA TTT GTC CTC A--3' and 5'--CCA TCC AAC ATC TCA GCA TGA TGA AA--3'). Resultant amplicons were purified (QIAquick PCR Purification Kits, Qiagen), sequenced with the direct method (ABI Prism 3700 DNA Analyzer, Applied Biosystems), and resultant sequences were subjected to nucleotide BLAST in GenBank to associate amplicon sequence with known vertebrate cytochrome B sequences (http://www.ncbi.nlm.nih.gov/blast/Blast.cgi).

### Bed net use, house characteristics and malaria vectors

To describe effect of bed net use and house characteristics on abundance of malaria vectors, a bed net use and house characteristics data set was generated (that is analysed in depth separately). These data include bed net ownership per house, as well as per individual spaces within the house. The available spaces in the houses included bedrooms, corridors, storage areas, sitting rooms and kitchens. The data included overall number of spaces in the house from which mosquitoes were collected, number of people who slept in each space and how many used bed nets, whether people slept on beds or on mats and the number of mosquitoes collected from each space. For this data set, due logistical reasons (no dissecting microscope in field and time constraints), mosquitoes were identified only broadly, as either anophelines or culicines. Additionally, house roofing and wall materials as well presence or absence of eaves were recorded.

### Climate and weather

During the 2009-10 study period, temperature and relative humidity were collected continuously using 10 temperature data loggers (HOBO, Onset Computer Corporation, Bourne, MA, USA) in the 8 study villages. The data loggers were suspended at vantage positions on the eaves of houses for easier access from outside when offloading. Daily rainfall data were collected using 4 HOBO event data logger rain gauges (HOBO, Onset Computer Corporation, Bourne, MA, USA) that are located within the study villages in the three study districts. For the 1997-1998 study period, rainfall data from 1993 to 2010 were obtained from Mtwapa meteorological station located ~30 km from the study districts. To compare rainfall trends during the two study periods, annual and seasonal rainfall data were compared between the 1993-1999 and the 2000-2010 periods using a *t *test. The 1997 rainfall data were excluded from the comparisons because of exceptionally high rainfall due to an El Niño event.

### Data analysis

Data were analysed using Statistical Analysis Software (SAS) Version 9.1 (SAS Institute). Human biting rate (HBR) was calculated by dividing the number of blood fed and half fed mosquitoes per house by the total number of people who slept in that house the night prior to mosquito collection. Human blood index (HBI) is the proportion of blood meals taken on humans while sporozoite rate (SR) is the proportion of mosquitoes with sporozoites in their salivary glands. The entomological inoculation rate (EIR) was approximated by multiplying the mean species HBR over the entire study period (639 days) by the overall species-specific SR to estimate the mean number of infective vectors per person. This value was multiplied by the species-specific HBI to estimate the mean number of infective vectors that would have inoculated humans for the entire study period. Chi-square and Fisher's exact tests were used (as appropriate) to compare the differences in the human blood index (HBI), SR and HBR of *An. gambiae *s.l. and *An. funestus *between quarters (seasons) and study periods. For 1997-1998, the mean numbers of *An. gambiae *s.l. and *An. funestus *per house for the entire study duration were calculated from data provided by CM [27]. The EIR for 1997-1998 was from Mbogo and others [27] while *An. gambiae *s.l. sibling species proportion data was from Mwangangi and others [40]. Statistical significance of the effect of presence of bed net, cumulative weekly rainfall with a lag of 3 weeks and mean weekly temperature with a lag of 3 weeks on the abundance of *An. gambiae *and *An. funestus *was tested by Poisson regression using the GENMOD procedure in SAS. Presence of bed nets was a categorical (dichotomous) variable (present/absent). The temperature and rainfall variables were continuous variables. A similar Poisson analysis for HBR was repeated where abundance of female mosquitoes was substituted by human biting rate per person per house, with the other variable remaining the same. Separate models were generated for *An. gambiae *and *An. funestus*.

#### Bed net use and house characteristics data

Wall materials of PSC sprayed houses were categorized broadly into mud walls (mud, thatch and grass walls) and block walls (walls made of blocks, stones, bricks and corrugated iron sheets). Roofing materials were also categorized into two broad groups; iron sheet roofs (corrugated iron sheets and tile roofs) and thatch roofs (roofs made of palm tree leaves and grass). Houses were classified as well-constructed (with block walls, iron sheet roofing and no eaves) or poorly-constructed (all other combinations of wall and roof materials, and presence of eaves). Well-constructed houses were considered well screened for preventing mosquito entry. Spaces where nobody slept the night prior to PSC were collectively grouped as "non-sleeping areas" while all spaces where someone slept were grouped as "sleeping areas". The term "bed type" as used here refers to either beds or mats, the types of sleeping equipment people use to sleep on. In the sleeping areas, bed net use was categorized as: 1) "Net used"- everyone who slept in that sleeping space used a bed net the night before mosquito collection, 2) "Net not used"- none of the people who slept in the sleeping spaces used a bed net even though bed nets were available the night before mosquito collection, or 3) "No net"- bed nets were not available to any of the persons who slept in the sleeping spaces the night before mosquito collection. Factors determining abundance of malaria vectors were tested by Poisson regression using the GENMOD procedure in SAS. In the model, the response variable was the log number of mosquitoes per sleeping space while the explanatory variables were net use (net used or net not used) house construction (good or poor), bed type (mat or bed), number of people per sleeping space (1-3 people versus > 4 people) and presence or absence of domestic animals (cattle, sheep, goats and donkeys).

### Ethical clearance

Verbal consent was obtained from the household head or their representative before commencing mosquito collection. These mosquito surveys were performed under human investigations protocols approved by the Ethical Review Board of Kenya Medical Research Institute (Nairobi, Kenya) and by the Human Investigations Review Board of Case Western Reserve University Hospitals (Cleveland, OH) and Emory University (Atlanta, GA).

## Results

### Climate and weather

**Figure 2:**
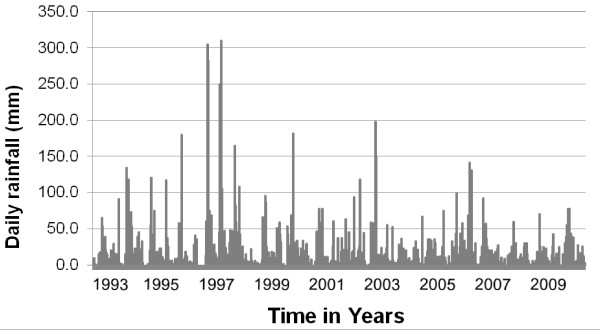
**Daily rainfall at Mtwapa meteorological station, 1993-2010**.

**Figure 3:**
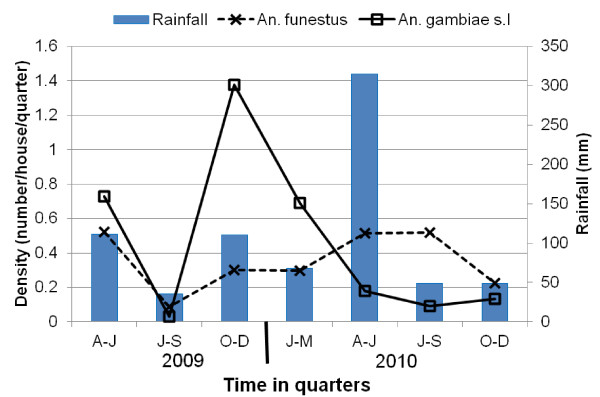
**Temporal variation in density of female resting *An. gambiae s.l*. and *An. funestus***.

Rainfall over the entire coastal Kenya is typically bimodal, with a long rainy season in March-June and a shorter rainy season that is much less predictable in October-December. A cooler, dry season, also highly unpredictable, occurs in July-September and the hot and dry season is usually between January and March. In the 2009-2010 study, both rainy seasons in 2009 were below average [41]. The short rainy season in 2010 also failed, but the total annual rainfall in 2010 was above average and much higher than in 2009 (Figure 3). Relative humidity was highly correlated with rainfall, and ranged from 72% to 79% over the study period. The annual rainfall for 1997 and 1998 was 3,267.7 mm and 1,391.5 mm respectively; in contrast, the amount of rainfall during the 1997-98 study period (June 1997-May 1998) was 2,943.2 mm. During 1997 and 1998, average rainfall fell during the long rains, but more rain fell during the short rains than the long rains in 1997, while the short rains failed in 1998. The average rainfall for the 18-year period of 1993-2010 was 1,431.0 mm (1,323.0 mm excluding the El Niño year of 1997). Comparison between 1993 and 1999 (excluding 1997) and 2000-2010 using a *t *test showed no significant differences for the long rains, short rains or the cooler and dry seasons (July to September). Although during the long dry season (January-March), more rain was recorded in 2000-2010 compared to 1993-1999, this difference was not significant (*P *> 0.05). There were two clear drought cycles during the 2000-2010 period [41], but no easily discernable ones in 1993-1999 (Figure 2).

### Mosquito abundance and human biting rate

**Figure 4:**
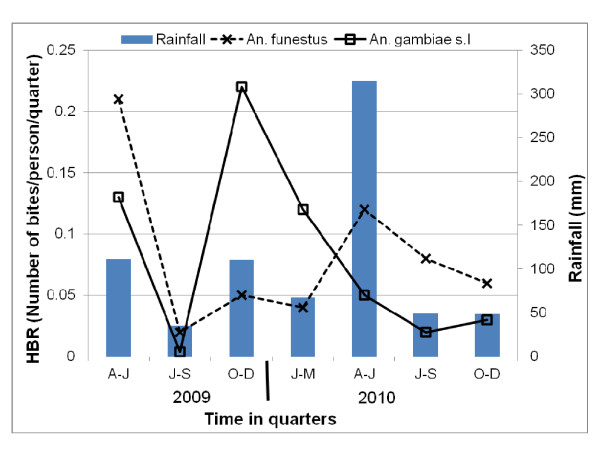
**Temporal variation in human biting rate of resting female *An. gambiae s.l*. and *An. funestus***.

A total of 43,973 mosquitoes from 1,777 houses were collected indoors by PSC and outdoors using clay pots in the 21-mo period. Culicines were collected most frequently (95.1%) followed by *An. gambiae *s.l (2.6%) and *An. funestus *(2.2%). *Aedes *spp. accounted for only 0.1% (45 individuals) of the total number of mosquitoes collected. During 17 months of mosquito collection, clays pots had collected very few mosquitoes (18 *An. funestus *and 60 *An. gambiae s.l.; *Table 1) and were therefore discontinued. Relative to PSC, clay pots collected lower proportions of females than males and also lower proportions of fed and gravid mosquitoes for both *An. gambiae *s.l. and *An. funestus*. However, clay pots collected higher proportions of unfed mosquito of both species. Due to the poor catch by clay pots, all the analyses here combined mosquitoes collected by PSC and clay pots. Among the 2,100 malaria vector collected, 963 were *An. funestus *and 1,137 were *An*. *gambiae *complex, of which 603 (53.0%) were processed by PCR (Table 1). The number and the relative proportion of both vector species varied during the 21-mo period, with *An. gambiae s.l*. much more abundant in 2009, while *An. funestus *dominated in 2010 (Table 1 & Figure 3). The number of *An. gambiae *s.l. collected was highest during the short rainy season of 2009 (October-December 2009 quarter). Both species were at very low densities during July-September 2009 quarter. The number of *An. funestus *collected was highest during April-June 2009 quarter. Female resting densities (mosquitoes/house/quarter) ranged from 0.03 to 1.38 for *An. gambiae *s.l. and from 0.08 to 0.52 for *An. funestus*. The human biting rate corresponded to the seasonal mosquito density variations (Figure 4). In 1997-1998, PSC collections netted an average of 1.83 female per house for *An. gambiae *s.l and 4.27 for *An. funestus*. In contrast, only 0.46 *An. gambiae *s.l and 0.36 *An. funestus *females were collected per house in 2009-10 (Table 2). A reduction of 75% and 92% was estimated, respectively, for *An. gambiae *s.l and *An. funestus *densities. Estimated reductions in HBR were 85% and 91% respectively for *An. gambiae *s.l and for *An. funestus*.

**Table 1:** Number and percentage of culicines and anophelines collected between April 2009 and December 2010 using PSC and clay pots in south coast, Kenya

	PSC (indoor)	Clay pots (outdoor)	Total
Culicines	41,453	373	41,826

*An.funestus *	945	18	963

*An.gambiae *s.l.	1,078	60	1,137

Total anophelines	2,023	78	2,100

No. *An.gambiae *s.l. tested by PCR	572	31	603

% *An. arabiensis *	86.0	89.0	86.0

% *An. gambiae*	14.0	11.0	14.0

**Table 2:** Mean number of mosquitoes per house (95% CI) and EIR in 2009-10 and 1997-98 and the percent decline in house mosquito densities and EIR

	2009-2010	1997-1998	% decline
Mosquito density (number/house)			

*An. gambiae *s.l	0.46 (0.30-0.62)	1.83 (1.39-2.26)	75%

*An. funestus*	0.36 (0.27-0.44)	4.27 (3.08-5.47)	92%

Both	0.82 (0.63-1.01)	6.10 (4.73-7.48)	87%

Entomological inoculation rate (EIR)			

*An. gambiae *s.l	0.15	20.44	99%

*An. funestus*	0. 29	11.68	97%

Both	0.43	32.12	99%

Data for 1997-1998 was recalculated from [27]

Overall, at least one bed net was found in 86% (1531/1777) of the houses where PSC was conducted. The mean number of bed nets per house was 1.8 nets, with an average of one net per 2.5 persons. The overall mean density of female *An. funestus *was 0.32 (CI at 95% = 0.24-0.41) in houses owning bed nets and 0.56 (CI at 95% = 0.19-0.93) in houses that did not own bed nets. By Poisson regression, houses that did not own bed nets had higher densities of female *An. funestus *(*P *< 0.01) relative to those owing bed nets. Poisson regression results also showed that abundance of *An. funestus *was not associated with the 3 weeks lagged weekly cumulative rainfall (*P *> 0.1), nor with the 3 weeks lagged mean weekly temperature (*P *> 0.3). For *An. gambiae *s.l. people owning nets were exposed to higher mosquito densities (0.47, CI at 95% = 0.29-0.65) compared to people not owning nets (0.38, CI at 95% = 0.16-0.60). Poisson regression for *An. gambiae *s.l., showed no difference in degree of protection for people living in houses with nets when compared to people living in houses without nets (*P *> = 0.4). Unlike with *An. funestus*, *An. gambiae *s.l abundance was sensitive to 3 weeks lagged weekly cumulative rainfall (*P *< 0.01) and average weekly temperature (*P *< 0.0001). Relatively few houses accounted for most *An. gambiae *s.l mosquitoes, resulting in most houses having none, i.e. 10 houses contributed 44% of all female *An. gambiae *s.l. mosquitoes. When these 10 houses were removed, the mean number of female *An. gambiae *s.l. mosquitoes dropped to 0.25 (CI at 95% = 0.20-0.31) in houses owning nets and to 0.31 (CI at 95% = 0.14-0.48) in houses not owning nets. With the 10 houses removed, there was no significant difference in protection levels (Poisson regression, *P *> 0.1) while the association with rainfall (*P *< 0.0001) and temperature (*P *< 0.0001) were highly significant.

### Relative densities of *An. gambiae *s.s. and *An. arabiensis*

Based on PCR, *An. gambiae *s.l included both *An. arabiensis *and *An.gambiae *s.s. in PSC and clay pot collections (Table 1). *Anopheles arabiensis *was the predominant species collected by both PSC (86%) and clay pots (89%). The proportions of the two species by the two sampling methods were not statistically different (*χ*^2 ^= 0.18, df = 1, *P *> 0.5). This is a marked decrease in *An. gambiae s.s*. and a proportionate increase in *An. arabiensis *in the study area compared to 12 years earlier when bed net use was negligible (Table 1). In 1997-98, *An. gambiae *s.l. comprised 81.9% (1606/1961) *An. gambiae *s.s. and 12.8% (251/1961) *An. arabiensis*, translating to a proportional decrease of 83% in *An.gambiae *s.s and an increase of 85% in *An. arabiensis *from 1997/98 to 2009/10 (Table 1). The total number of anopheline larvae sampled was 520 in 1,104 sampling efforts and fluctuated with seasons. The mean number of larvae per quarter ranged from 0.32 in January-March 2010 to 1.07 in April-June 2009. At the larval stage, the decline in *An. gambiae s.s *was of the same magnitude as for the adults between the two study periods. Of the 73 *An. gambiae s.l*. larval samples subjected to PCR, 82% (60) were successfully processed and 85% (51) were identified as *An*. arabiensis with the remaining15% as *An. gambiae s.s*. (Table 3).

**Table 3:** Proportion of *An.gambiae *s.l larvae from the study area that were identified as *An. arabiensis *or *An. gambiae *s.s. in 2009-2010 and 1999

	2009-2010	1999
No. of *An.gambiae *s.l. larvae tested by PCR	60	377

% *An. gambiae *s.s.	15	86

% *An. arabiensis*	85	14

Data for 1997-1998 was from [34]

### Blood meal identification

A total of 141 *An. gambiae s.l*. and 91 *An. funestus *were tested for blood meal source (Table 4). The success rate in PCR, sequencing, and BLAST match was high. Most individuals (87.8%) of both species had fed on human beings. A significantly higher proportion of *An. gambiae s.l*. fed on cattle and goats when compared to *An. funestus*. Blood meal identification results of the current study and of a study conducted in the same area in 1997-1998 [40] are shown in Table 4. The proportion of *An. gambiae s.l*. feeding on humans was reduced by 14.7% and for *An. funestus *by 5.5%. The proportion of mosquitoes feeding on cattle for both *An.gambiae s.l*. and *An. funestus *increased by 100% and on goats by 84.6% for *An. gambiae s.l*. and by 57.1% for *An. funestus*. Given that the majority of the *An. gambiae s.l*. were *An. arabiensis *(86%), the HBI for *An. arabiensis *is estimated to be very high (over 80%).

**Table 4:** Blood-meal sources for *Anopheles gambiae *s.l. and for *Anopheles funestus *during 1997-1998 and 2009-2010 in south coast, Kenya

Study period	Species	Tested	Blood meals				
			
			Human	Cattle	Chicken	Goat	Mouse
1997-1998	*An. gambiae *s.l.	312	238 (98.4)	0 (0.0)	1 (0.4)	3 (1.2)	0 (0.0)
	
	*An. funestus*	244	196 (99.5)	0 (0.0)	0 (0.0)	1 (0.5)	0 (0.0)
	
	**Total**	**556**	**434 (98.9)**	**0 (0.0)**	**1 (0.2)**	**4 (0.9)**	**0 (0.0)**

2009-2010	*An. gambiae *s.l.	141	115 (83.9)	11 (8.0)	0 (0.0)	11 (8. 0)	0 (0.0)
	
	*An. funestus*	91	79 (94.1)	3 (3.6)	0 (0.0)	1 (1.2)	1 (1.2)
	
	**Total**	**232**	**194 (87.8)**	**14 (6.3)**	**0 (0.0)**	**12 (5.4)**	**1(0.5)**

Data for 1997-1998 was derived from [40]

### Malaria infection

174 *An. funestus *and 292 *An. gambiae s.l*., which had been collected between August 2009 and September 2010, were tested. Among the 292 *An. gambiae *s.l., 202 were *An. arabiensis*, 23 were *An. gambiae *s.s and 67 had not been identified to sibling species by PCR. Overall, only two mosquitoes were positive for sporozoites from two different villages, one *An. arabiensis *collected in December 2009 and one *An. funestus *collected in July 2010. The sporozoite rate was 0.0057 in *An. funestus*, 0.0034 in *An. gambiae *s.l. and 0.0043 from all potential vectors. The overall EIR over the study period of 21 months was 0.43 infective bites/person and 0.29 infective bites/person for *An. funestus *and 0.15 infective bites/person for *An. gambiae *s.l. This represented a 99% reduction in malaria transmission potential compared to 1997-98 (Table 2).

### Bed net use, house characteristics and malaria vectors

Pyrethrum spray catches were conducted in a total of 5,318 spaces within 1,571 houses. More than half (57%; 3,044/5,318) of these spaces were sleeping areas, with the remaining 43% (2747/5318) non-sleeping areas. In total 1,845 malaria vectors were collected; 70% (1,289) from the sleeping areas and 30% (556) from the non-sleeping areas. Bed net use was not compared between the different types of sleeping places because very few people (3%) slept away from bedrooms in places such as kitchens or sitting rooms. In sleeping areas, bed nets were used by all in 68.3% (2,079/3,044) of the spaces, were available, but not used by anyone in 7.7% (234/3044) of the spaces, and were not available to anybody in 731 (24.0%) of the spaces (Table 5). We excluded from this analysis all spaces where some people slept on beds and others on mats (n = 76, 2.5% of total spaces), or where only some people used bed nets (n = 67, 2.2% of total spaces). The Poisson regression model indicated that besides bed net use, malaria vector abundance was also positively associated with the use of mats for sleeping and with poorly screened houses (Table 6). Presence of domestic animals was positively associated with higher mosquito densities. Higher densities of malaria vectors were found resting in sleeping places where a sleeping mat was used compared to those where a bed was used, irrespective of whether a bed net was used or not (Table 7). People sleeping on mats were less likely to use nets (net was used in 45% of the sleeping places where a mat was used) compared to those sleeping on bed (70% net use). Moreover, only 31% of houses where a mat was used were well constructed compared to 44% of houses where a bed was used.

**Table 5:** Number and proportion of sleeping places that used nets (76 sleeping places with both beds and mats and a further 67 spaces where only some used bed nets were excluded)

	Beds	Mats	Total
**Net use**			

Net used	**1,868 (70.6%)**	**113 (44.5%)**	**1,981 (68.3%)**

Net not used	193 (7.3%)	31(12.2%)	224 (7.7%)

No net	586 (22.1%)	110 (43.3%)	696 (24.0)

Net not used or absent	**779 (29.4%)**	**141 (55.5%)**	**920 (31.7%)**

the last row is a summary of non-bed net use (rows 2 and 3)

**Table 6:** Abundance of malaria vectors in south coastal Kenya in relation to bed net use, house characteristic and presence or absence of domestic animals based on Poisson regression

Bed net use and house characteristics	Parameter estimate	Lower and upper 95% CI	Wald chi square	*P *value
***Bed net use***				

Net not used (68%)	0.54	0.25-0.83	13.34	0.0003

Net used (32%)	-	-	-	-

***Bed type***				

Mat (9%)	0.88	0.54-1.22	26.40	< 0.0001

Bed (91%)	-	-	-	-

***House construction***				

Poor (42%)	1.07	0.72-1.44	34.04	< 0.0001

Good (58%)	-	-	-	-

***Domestic animals***				

Yes (35%)	0.43	0.15-0.71	9.30	< 0.01

No (65%)	-	-	-	-

**# of people per sleeping space**				

1-3 (60%)	0.13	-0.16-0.42	0.80	> 0.3

Over 3 (40%)	-	-	-	-

**Table 7:** Total number and mean (CI at 95%) of indoor resting anophelines in sleeping places with beds or mats by net use

Net use	Beds		Mats		Total
	**Total^a ^**	**Mean (CI)^b^**	**Total^a^**	**Mean (CI)^b^**	

Net used	574	0.31 (0.22-0.40)	65	0.57 (0.23-0.92)	639

Net not used or absent	351	0.45 (0.27-0.62)	210	1.49 (0.82-2.16)	561

Total	925	0.35 (0.27-0.43)	275	1.08 (0.68-1.48)	1,200

^a^total number of malaria vectors per bed net use category

^b^mean number of malaria vectors per bed net use category

## Discussion

Bed net use in most malaria endemic countries, especially in sub-Saharan Africa, has risen to high levels during the last 5-7 years [42-44]. Most published reports describe the impact of bed net use on malaria vectors under situations where bed net use is studied as part of a controlled [11,12,45,46] or partially controlled [13,23] experimental field study, with the duration of bed net use ranging from 1 to 3 yrs. This report examines the association of bed nets with a decline in malaria mosquito vectors and on their effectiveness under a national bed net distribution programme and over a long period of use (over 5 years). A remarkable decline in indoor resting densities, and corresponding human contact of the two most important malaria vectors, is reported; *An. funestus *and *An. gambiae s.l*., showed a significant decline in the relative proportion of *An. gambiae s.s*. among collected mosquitoes, coupled with a proportionate increase of *An. arabiensis *and a shift towards non-human vertebrates feeding in both *An. funestus *and *An. gambiae s.l*. after 5-7 years of high bed net use in southern coastal Kenya. Overall, the 85% reduction of indoor mosquito densities since 1997-8, with a wider reduction in *An. funestus *(12 times lower in 2009-2010) compared to *An. gambiae s.l*. (four times lower in 2009-2010) is coincidental with a decade of notable increase in ownership and use of bed nets [28-30].

The marked decline in malaria vector densities resulted in very low intensity of malaria transmission as measured by EIR. In contrast to 1997/1998, throughout much of the study period transmission was undetectable and highly seasonal [27]. The reductions in entomological indices corroborate recent observations on human malaria prevalences in the study area and in Coast Province in general. Malaria prevalence in school children has declined from 64.3% in 1997-1998 [27] to an estimated range of 2.3% -14% [47,48]. Other reports have pointed to notable decreases in malaria hospitalization that coincided with scaled bed net intervention and a shift in malaria drug policy [6,31].

The reductions in entomological indices reported in this study occurred in a setting where people who use neither ITNs nor untreated nets daily reside in same villages and houses with people who either do not own nets or who rarely use them even though they own them. The significant differences in vector densities between protected and unprotected persons for *An. funestus *strongly suggest that reductions of both the indoor mosquito densities and their associated human contact are mainly due to a long-term increase in bed net use over a period of > 5 years. Intensive use of bed nets over short [13,22] and long periods of time were previously associated with changes in the ratio of *An. gambiae *s.s to *An. arabiensis *both at larval and adult stages [23]. The striking decline in *An. gambiae *s.s density and the corresponding increase in density of *An. arabiensis *in the study area is a clear affirmation of the effectiveness of bed nets. The increased proportions of the exophagic, zoophagic *An. arabiensis *explain the lack of complete protection for individuals owning nets. The conclusion that much of the decline in malaria vectors is a result of prolonged community-wide bed net use is further sustained by the slight shift to more non-human feeding in this study as demonstrated elsewhere [24-26].

Even though the data presented here overwhelmingly suggest that the decline in indoor resting mosquitoes is most likely due to sustained bed net use, other reasons cannot be ruled out. In agreement with previous observations that showed poor house design as a risk factor for malaria [49-56], people living in better constructed or 'screened' houses were more protected. Mud walls, thatch roofs and open eaves provide more opportunities for mosquito entry and resting points when compared to concrete walls and iron sheet roofs [54,55]. Presence of domestic animals is predicted to be a malaria risk factor because only a small proportion of the malaria vectors were found to have fed on domestic animals. When livestock is kept very close to houses and sometimes inside the houses as is the case in the study area, they may attract mosquitoes to the houses, but once inside the house the mosquitoes could preferentially feed on humans [52,54,55]. In the study area, the few residents sleeping on sleeping mats on the ground are much more exposed to anophelines than those sleeping on beds, regardless of the use of bed nets. This is because people sleeping on mats are less protected by nets (when used) and often live in poorly constructed houses. The results suggest that consistent with previous findings, effectiveness of bed nets is greatly confounded by household socioeconomic status [54,57]. The 1997-1998 study did not collect data on house characteristics; however it is implausible that there have been significant gains in structural improvement of houses or in socioeconomic status over the last 10 years that resulted in indoor resting mosquito density declines of the magnitude reported here [58].

During the 2009-10 study period, an attempt was made to collect outdoor resting mosquitoes but there was no comparable effort during the 1997-8 study period. The failure of clay pots as an outdoor mosquito sampling tool remains unexplained. A previous study that used clay pots both indoors and outdoors attributed poor catch outdoors to dry and hot weather in northern Tanzania [59] and this may be the case at the Kenyan coast where temperatures are relatively high compared to Western Kenya where clay pots were very efficient [36]. Besides quantifying the outdoor mosquito densities, clay pots were used to provide the evidence that prolonged bed net at high coverage resulted in more outdoor resting [21,22]. Lack of information on exophagy is therefore a major limitation of the current study. Though not clearly elucidated by the data presented here, the slightly more variable rainfall patterns experienced during 2000-2010 period [41] could have contributed to the declines in mosquito densities, as shown elsewhere [60].

Despite the seasonal fluctuations in densities of *An. gambiae s.l*., especially in 2009, both *An. gambiae s.l*. and *An. funestus *occurred at almost equal frequency and all the entomological indices studied point to both vectors being equally important in malaria transmission. *An. funestus *contributed the most to malaria transmission in 1997/1998 [27] and persists as the most important vector in the study area. *Anopheles funestus *is likely to continue as the more important long-term vector of malaria under prevailing circumstances. *Anopheles funestus *population is reported to have crashed under intensive bed net use even over a short time [11,24,45], but this was not observed in the present study area, despite more than five years of high level bed net use. The presence of more than thirty-two percent unprotected persons, coupled with the sustained output from more stable breeding sites (Mutuku FM, unpublished data), [34]), explain the persistence of *An. funestus *despite considerable enduring intervention pressure. The fact that both personal protection and community-wide effects were not sufficient to diminish the role of *An. funestus *highlights the significance of the interaction between local mosquito ecology and bed net coverage.

In conclusion, the results presented here suggest that further suppression of *An. funestus *population in the study area is feasible with sustained bed net use at higher levels than the current ones. Alternative and/or supplementary vector control methods may however be required to supplement bed nets for *An. arabiensis*. These findings also point to the need for similar longitudinal studies at local and regional levels cognizant of the historically heterogeneous nature of malaria transmission [27,61-63] and varying levels of bed net use [30,44]

## Competing interests

The authors declare that they have no competing interests.

## Authors' contributions

FMM, CHK, PM, EMM, EDW, and UK designed the study and wrote the manuscript. FMM, CHK, PM and UK collected and processed mosquitoes; EDW identified mosquitoes of the *A. gambiae *s.l. complex and mosquito blood meals with PCR. CM and JM contributed data and performed ELISAs for mosquito malaria infection. All authors read and approved the final manuscript.
